# Drug dosing in children with obesity: a narrative updated review

**DOI:** 10.1186/s13052-022-01361-z

**Published:** 2022-09-08

**Authors:** Francesca Gaeta, Valeria Conti, Angela Pepe, Pietro Vajro, Amelia Filippelli, Claudia Mandato

**Affiliations:** 1 Pediatrics Section, Department of Medicine, Surgery and Dentistry “Scuola Medica Salernitana”, University of Salerno, 84081 Baronissi, Salerno Italy; 2 Pharmacology Section, Department of Medicine, Surgery and Dentistry “Scuola Medica Salernitana”, University of Salerno, 84081 Baronissi, Salerno Italy

**Keywords:** Children, Obesity, Drugs, Drug dosing, Clinical practice, pharmacodynamics, pharmacokinetics, therapy, pharmacology

## Abstract

Childhood obesity and its associated comorbidities are highly prevalent diseases that may add to any other possible health problem commonly affecting the pediatric age. Uncertainties may arise concerning drug dosing when children with obesity need pharmacologic therapies. In general, in pediatric practice, there is a tendency to adapt drug doses to a child’s total body weight. However, this method does not consider the pharmacological impact that a specific drug can have under a two-fold point of view, that is, across various age and size groups as well. Moreover, there is a need for a therapeutic approach, as much as possible tailored considering relevant interacting aspects, such as modification in metabolomic profile, drug pharmacokinetics and pharmacodynamics. Taking into account the peculiar differences between children with overweight/obesity and those who are normal weight, the drug dosage in the case of obesity, cannot be empirically determined solely by the per kg criterion. In this narrative review, we examine the pros and cons of several drug dosing methods used when dealing with children who are affected also by obesity, focusing on specific aspects of some of the drugs most frequently prescribed in real-world practice by general pediatricians and pediatric subspecialists.

## Introduction

The World Health Organization has declared childhood obesity “a serious global health pathological condition and an evolving emergency of the 21st century”, with an estimated 124 million children with obesity worldwide [1, 2]. There are large differences among countries, with Southern Europe having a higher number of children with overweight, compared to Northern Europe. Moreover, industrialized countries tend to have more awareness about obesity-related risks than less-developed or middle-income countries [3].

In addition to the usual large cluster of childhood illnesses, children with obesity may have to face also a number of weight-related specific comorbidities that formerly were typical of adult age, mostly under the umbrella term of metabolic syndrome (i.e. hypertension, hypercholesterolemia, type 2 diabetes), and also fatty liver, obstructive sleep apnea, polycystic ovary syndrome, and cancer [4].

In general, doses of drugs are scaled based on the individual patient characteristics, including age, weight, and comorbid conditions. Regarding weight, several parameters are taken into consideration, amongst them, there are: total body weight (TBW), which represents the actual weight, and ideal body weight (IBW) based on age, weight and height. The simple adaptation of doses used in adults to children’s TBW does not consider the effects of a specific drug across different ages and weight groups. In children with obesity, drug administration based on TBW may result in overdosing, while, on the other hand, the use of of IBW may lead to underdosing.

Lean body weight (LBW), based on the difference between TBW and fat mass, technically is the best weight scalar for drug administration, but it is difficult to accurately measure.

A further relevant challenge may depend on modifications of pharmacokinetic (PK) parameters. Among them, the volume of distribution (Vd) and clearance (CL) are the most affected by the clinical condition of overweight or obesity. Vd (measured in L) is a theoretical concept used to relate the total amount (dose) present in the body to the one present in the body fluids. CL (measured in L/h or mL/min) represents the volume of plasma from which a drug is completely removed per unit time. The total body clearance is calculated by the sum clearance of the drug by each organ such as kidney, liver, and lung. Understanding how the Vd and CL vary in children with overweigh/obesity compared to peers with normal weight is crucial since the first determines the selection of drug loading-dose and the second one the drug maintenance dose regimen.

Modifications in these PK parameters as well as other obesity-related alterations, including those in drug absorption and metabolism, might affect the drug therapeutic index, that is a ratio comparing the blood concentrations at which a specific drug exerts its therapeutic effect to those associated to occurrence of toxicity [5, 6]. Moreover, in children and adolescents with obesity the effect of gender on drug absorption and metabolism are rarely considered in spite of gender differences in obesity-related metabolic syndrome clustering before and after puberty [7], and sex related differences in gastrointestinal physiology and intestinal microbiome [8].

In June 2020, Ameer et al. [5] published a comprehensive review of the drugs most widely used for the treatment of diseases in infants, children, and adolescents with obesity. The reviewed literature, covering the period from 1970 to 2018, focused on drugs used in hospital and highlights that the enrolled cohorts are often small, and large variability on methods was used for drug dosing. To provide an update and an extension of Ameer’s data [5], here we reviewed the existing recommendations and suggested methodological approaches for drug dosing in children with obesity considering drugs commonly prescribed both in- and out-of-hospital settings.

## Methods

The topic area and the population of interest for the research question were “drug dosing” in “children with obesity” or “obese children”, respectively. To these aims we searched the inherent English literature of the last 11 years appearing in PubMed and Scopus databases. The database search strategy was formulated around terms “child” and several other text words chosen based on the existing literature and/or obtained from related bibliographies, combined using Boolean operators (obesity; obese; drugs; drug dosing; pharmacodynamics, pharmacokinetics, therapy, pharmacology, and the name of specifically selected drugs). A manual search of relevant articles cited within the retrieved studies was performed as well. The earliest publication date was January 2010, and the search ended in December 2021. Information about dosing in pediatric obesity extracted from each of the articles was identified as relevant to the review if it concerned a drug habitually used in pediatric clinical practice, also based on a literature comprehensive list of the most commonly prescribed drugs to children [2].

### Generalities on the methods proposed for dosing drugs in children with obesity

Therapeutic drug dosing methods used in children with obesity are based on simple body weight-based dosing and several other weights [e.g. TBW, IBW, body mass index (BMI)], age, allometric scale, fat-free mass related measures. Furthermore, they also include surface area and physiologically based dosing (particularly clearance-based dosing) [2], potentially leading to the risk of either over- or under-dosing) [9]. Table 1 describes the main methods and summarizes the pros and cons of each of them. Globally, it appears reasonable to establish the drug dosing using methods accurately based on calculations that consider both the characteristics of the child, the physiological consequences of obesity, and also a possible increased toxicity due to the individual patient susceptibility [10]. Familiarity with adult dosage regimens is also needed; recently, the Pediatric Pharmacy Advocacy Group [11] recommended that, in the absence of specific studies, compliance with the following key points may be useful in empirically determining medication dosage in children with overweight/obesity:

- weight-based dosing should be used in patients aged < 18 years who are < 40 kg,
- weight-based dosing should be used for children who are ≥40 kg, unless the patient’s dose or dose per day exceeds the recommended adult dose for the specific indication,
- familiarity with adult dosage regimens is needed to avoid exceeding the recommended maximum adult dose,
- Clinicians should consider the occurrence of modifications of PK parameters for adjusting drug dosage whenever possible in children with overweight/obesity to ensure the most effective and safe regimen [11].

**Table 1:** Methods for drugs dosing applied to children with obesity

Body Descriptor/ Equation/ Population	Remarks
**TBW** TBW: drug dose *kg Pediatric (up to 40 kg and 12 Years)	Not recommended for lipophilic drugs. For unfractionated heparins a lower dose is recommended [12].
**BSA** BSA (m2) = square root of (height (cm) x weight (kg)/3600) Children and adults	This method is used for both adults and children especially for dosing chemotherapy drugs.
**IBW** IBW = [50th percentile weight (age)*height(cm)] Age 2-20 years	Accurate determination of IBW is important for the proper dosing of medications, such as acyclovir, digoxin, and morphine. There is no consensus on the most accurate calculation in children. The growth chart-based Moore’s method determines IBW by looking at the same weight percentile line as the child’s height percentile for that age.
**BMI** BMI chart for age (weight in kilograms divided by the square of height in meters). Age 2–20 years	A high BMI can be an indicator of high body fatness. Pediatric BMI should be correlated with growth curves and percentiles.
**Allometric scale** Dose in children = adult dose * (TBW of a child/70)^0.75^ Age 2–20 years	Drug dose is predicted using allometry, depending on the properties of the drug such as free fraction in adults, pharmacodynamics and binding proteins [13].
**Fat Free Mass** FFM = weight [kg] * [1 - (body fat [%]/ 100)] Adults	Fat-Free Mass (FFM) refers to all body components except fat. It includes water, bone, organs and muscle content with different measure for males and females.
**Age scaling** The dose is selected according to the child’s age using charts. Over six months age.	This method does not take into account the changes due to developmental growth that occurs within each age group (e.g., the hepatic metabolic capacity of an infant child is different from that of a neonate)
**Physiologically Based Dosing**	Based on pathophysiology and changes in obesity, drug binding or distribution volume. The adipose tissue succeeds in getting the lipophilic molecules, making them less available for therapeutic effect. The increase in the blood volume and cardiac output of the child affected by obesity and the alteration of plasma proteins create alterations in the distribution of drugs [13, 14]
**Clearance Based Scaling** • Dose for the child = adult dose *(CL in the child/CL in adults) • CL in the child = CL in adults * (TBW of a child/70) ^exp^ The fixed exponent of 0.75 is commonly used and predicts reasonably well for children older than 2 years of age (as used generally in allometric scaling) [15].	A. Clearance is a measure of the drug metabolism in the gut and liver and/or their renal elimination. B. Obesity is a predisposing factor for liver steatosis in both adults and children, involving reactions that require modification and therefore the elimination of drugs. CYP3A4 activity is reduced in obese patients. The Clearance based method takes into account renal function too in terms of Volume of distribution and clearance. Obesity can affect kidney enzyme functions

Adapted in part from Xiong Y, Fukuda T, Knibbe CAJ, Vinks AA. Drug Dosing in Obese Children: Challenges and Evidence-Based Strategies. Pediatr Clin North Am. 2017;64(6):1417–38 [16]

*BSA* Body Surface Area, *CL* Clearance, *FFM* Fat Free Mass, *IBW* Ideal Body Weight, *TBW* Total body weight

### Common-use and sub-specialty drugs dosing in pediatric patients with obesity

Many of the drugs that are routinely used in pediatric clinical practice have a well-established dosage. Unfortunately, in the presence of obesity, this *static* approach cannot be considered valid and/or safe. Below, and in Table 2, a number of drugs commonly used by general and sub-specialty pediatricians [2] are shown in relation to their use in pediatric patients with obesity and to available information retrieved by our search.

**Table 2:** Most common drugs’ dosing adjustment in children affected by obesity

Drug	TBW (Total Body Weight)	Use In Children Affected By Obesity
**ANTIBIOTICS**		
Amoxicillin/clavulanic acid (combination of amoxicillin, a β-lactam antibiotic, and potassium clavulanate, a β-lactamase inhibitor)	25–70 mg/Kg/die	The practice of capping the dose at the usual adult maximum did not seem to differ whether prescribing for children with obesity or for normal-weight children [17]. Use TBW dosing, possibly evaluating the posology based on the severity and site of infection **[**18**]**
Azithromycin (macrolide)	10 mg/kg/day	Underdosing the TBW: risk of overdose due to difficult elimination [19].
Cefazolin (first generation cephalosporine)	25-100 mg/kg/day	No dose adjustment for obesity [2, 5]
Ceftazidime (cephalosporine)	40 mg/kg IV q6h	The administration maximized the model-based probability of target attainment PTA in children and adolescents with obesity and GFR ≥ 80 mL/min/1.73 m^2^ [20]
Clindamycin (lincosamide)	20-40 mg/kg/day in three or four equal doses.	Recommended weight-based dosing in children with obesity [21, 22].
Ceftriaxone (third-generation cephalosporin)	50-100 mg/kg/day	TBW dosing has proven safe and effective in childhood obesity [23].
Linezolid (oxazolidinones)	10 mg/kg day, max 600 m	Weight-based dosing in children remains unclear. Data from adult patients suggest risks of linezolid underdosing in empirical antibiotic therapy of most resistant bacteria [19].
Meropenem (carbapenem)	20 mg/kg IV every 8 hours	Dosage adjustments based solely on body weight are unnecessary [24, 25, 26].
Trimethoprim/sulfamethoxazole (cotrimoxazole)	8 mg/kg/day trimethoprim	Patients with overweight/obesity may have decreased weight-normalized clearance and volume of distribution of the drug, so that should require higher absolute doses under recommended pediatric weight-based dosing regimens [2, 21, 24]
Vancomycin (glycopeptide)	20-40 mg/kg/day	No dose adjustment for obesity [20].
**ANALGESICS AND ANESTHETICS**		
Acetaminophen/Paracetamol	10–15 mg/kg/day every 4-6 h <12y	No significant differences in circulating acetaminophen concentrations after a 5-mg/kg (up to 325 mg) single oral dose administration in children with NAFLD In adults there are higher concentrations of hepatotoxic CYP2E1-mediated acetaminophen metabolites. Adults with obesity may not tolerate high doses due the overproduction of hepatotoxic acetaminophen metabolites [2].
Dexmedetomidine (selective a2-agonist)	1 μg/kg	No differences in the dosage required for sedation in children suffering from obesity and those with normal weight [27]. Rolle et al. have found in their study that lean body mass (LBM) is an appropriate dosing scalar for size in adult patients with obesity [18].
Fentanyl (opiate agonist)	1–2 μg/kg/dose IM	Lipophilic. Adjusted Body Weight (cofactor of 0.25) has been recommended [28]. Mortensen et al. recommended TBW for induction and lean body weight (LBW) for maintenance of anesthesia [28].
Midazolam (benzodiazepine)	0.1–0.3 mg/kg, max 5 mg IV,IM	Potential need for higher initial drug dose administration for continuous infusion [29].
Morphine (opiate agonist)	0.1–0.15 mg/kg/dose every 4 h IM or 0.2–0.4 mg/kg/dose every 4 h OD	Dosing morphine is based on IBW because it is a hydrophilic opioid [28]. TBW not recommended.
Propofol (short-acting, lipophilic intravenous general anesthetic)	1–2 mg/kg pro dose	Diepstraten et al. proposed TBW-based dosing to achieve maintenance anesthesia [30].
**OTHER DRUGS**		
Amlodipine (Calcium channel blocker)	0.1 mg/kg/day	TBW [31]
Angiotensin-Converting Enzyme Inhibitor (Ramipril)	0.05–0.15 mg/kg/day max 40 mg/day	TBW based dose: an empiric low starting dose can be used [2, 32]
Antipsychotics (e.g. haloperidol, thioridazine, risperidone, aripiprazole)		Few studies on their correct dosing and therapeutic drug monitoring. Start low, go slow and careful monitoring of patients’ metabolism Discontinuation attempts after long-term use can also be beneficial [33, 34, 35].
Atorvastatin (statins)	> 10y: 10-20 mg/day	Due to the correlation of statins with the genotypic variability of SLCO1B1, cases of statin overtreatment may occur [36].
Antineoplastic drugs	Depending on the drug and protocols	Doses of chemotherapy are commonly calculated based on a patient’s Body surface area, using TBW. Baillargeon et al. by studying children with leukemia found that 7% of those with obesity received less than the protocol-specified dose [37].
Inhaled corticosteroids (e.g. beclomethasone, budesonide, flunisolide, fluticasone)	Depending on the drug	Standard doses are insufficient for children with obesity [14, 38]
Liraglutide (analogous to glucagon-like peptide)	Same dose of adults (i.e. 3 mg, s.c.).	Children over 10 years of age with an indication of type 2 diabetes mellitus not correctly compensated with metformin indications also for the treatment of obesity in patients aged> 12 years weighing > 60 kg
Low-molecular weight Heparin	Depending on the drug and indication	Dose adjusting of enoxaparin dosing on initiation of therapy is necessary [39, 40]
Metformin (biguanide)	500 mg day, max 2 g/day	TBW dosing: higher drug doses than patients without obesity [2] Use of adult doses of metformin in older children and adolescents with obesity [41]
PPIs e.g., Pantoprazole	10–20 mg/day	Dosing PPIs in obesity can be the same as for normal-weight children [5, 42]
Steroids	Depending on indication	Need for standardization of drug dosing guidelines for children with obesity to avoid risk of harm [38].
Vitamin D	1000 and 2000 IU 25(OH)D /day	The highest percentages of patients affected by obesity with values ≥20 ng/ mL were seen only among the 2000-IU group, implying therefore the superiority in effectiveness of this dose in comparison to the lower ones [43]

IBW = [50th percentile Weight (age)*height(cm)], *PPI* Proton Pump Inhibitors, *TBW* Total Body Weight, *LBM* Lean Body Mass, *NAFLD* Non Alcoholic Fatty Liver Disease

#### Antimicrobials

As for other drugs, dosing of antimicrobial agents merely based on TBW may be tricky in children with overweight/obesity. The Vd modifications are crucial in these patients who are likely overexposed particularly to drugs not adequately distributed into adipose tissue, while are underexposed to the agents easily distributed in the body tissues [24].

Amoxicillin/clavulanic acid (TBW dose 25–70 mg/kg) is a combination of amoxicillin, a β-lactam antibiotic, and the β-lactamase inhibitor potassium clavulanate. It is a hydrophilic antibiotic that is absorbed orally eliminated in the urine commonly administered to treat otitis media, group A strep throat, pneumonia, urinary tract infections, and animal bites. To our knowledge, no studies examining the pharmacokinetics of amoxicillin or other penicillins have been performed in children with obesity.

A retrospective cohort study, by considering the maximum prescribed dose for otitis media found that heavier children received lower doses of amoxicillin per kg of TBW. However, limiting the dose to the usual maximum adult dose did not appear to make any difference in terms of failure or relapse whether the prescription was for children with obesity or normal weight [18].

Macrolides The effect of macrolides against most bacteria is considered to be time-dependent due to a significant post-antibiotic effect, especially for azithromycin (AZM). While in adult patients with obesity standard doses are recommended, there are no data in pediatrics [19].

However, recent recommendations on off-label use of intravenous AZM in children suggest that when intravenous AZM is administered to children with obesity, the dosage calculated by body weight should not exceed the adult dosage (i.e. 500 mg per day) [44].

Cefazolin (TBW dose 25–100 mg/kg/day) is a first-generation cephalosporin used to treat cellulitis, urinary tract infections, pneumonia, endocarditis, joint infections, and biliary tract infections. Studies have not shown differences in cefazolin clearance or volume of distribution (Vd), considering TBW in children with obesity compared to those with normal weight, both in adults [45] and in a pediatric population [5].

Ceftriaxone (TBW dose 50–100 mg/kg/day) is a third-generation cephalosporin that is widely used for the treatment of many infections such as ear infections, endocarditis, meningitis, pneumonia, and bone and joint infections. TBW dosing has been shown to be safe and effective for childhood obesity [23].

Ceftazidime (TBW 100–150 mg/kg/day) PK was evaluated in a cohort characterized by a predominant number of children and adolescents with obesity. Administration of 40 mg/kg IV q6h (max 8 g/day) maximized the model-based probability of target attainment (PTA) in children and adolescents with obesity and GFR ≥ 80 mL/min/1.73 m2 [20].

Vancomycin (TBW dose 20–40 mg/kg/day) is a glycopeptide used for complicated skin infections, bloodstream infections, endocarditis, bone and joint infections, and meningitis caused by methicillin-resistant *Staphylococcus aureus* o*r Streptococcus pneumoniae*. Vancomycin is distributed into body tissues and fluids and is eliminated in urine. At pediatric age, the Vd for vancomycin varies from 0.26 to 1.05 L/kg, and the t½ varies from 6 to 10 hours in neonates and from 2 to 4 hours in infants and children. The absence of statistical differences in mg/kg TBW doses found between children with obesity, overweight, and normal weight suggests that there is no need for dose adjustment in case of obesity [46]. Serum concentrations, however, should be prudentially monitored, to avoid hearing and renal toxicity even if a recently proposed dosing guideline integrating TBW and creatinine clearance resulted in effective and safe exposures across all ages, body weight, and renal functions in the pediatric population with varying degrees of obesity and renal function [17].

Meropenem (60 mg/kg/day) is an effective and safe treatment for infants and children with severe pediatric infections, including bacterial meningitis. It is used also in patients with cystic fibrosis, and respiratory diseases at higher doses [24, 25]. A recent study of population pharmacokinetic modeling was performed using the Nonlinear Mixed-Effect Modeling (NONMEM) software and 5000-patient Monte-Carlo simulations to calculate PTA. A 2-compartment linear-elimination model best described the serum concentration-time data, and creatinine clearance was significantly associated with systemic clearance Meropenem pharmacokinetics resulted comparable among patients without or with obesity, including morbid obesity. Standard dosing regimens provide adequate pharmacodynamic exposures for susceptible pathogens at 40 and 54% fT > MIC (concentration of the antibiotic in the blood, times above the minimum inhibitory concentration), but prolonged infusions of larger doses are needed for adequate exposures at 100% fT > MIC. Dosage adjustments based solely on body weight are unnecessary [26].

#### Clindamycin and trimethoprim/sulfamethoxazole

Clindamycin (30 to 40 mg/kg/day) is a lincosamide antibiotic used to treat severe infections. Trimethoprim/sulfamethoxazole (TMP/SMZ) also known as co-trimoxazole (8 mg/kg/day trimethoprim) is a fixed-dose combination of two antimicrobial agents belonging to a class of medications called sulfonamides that act synergistically against a wide variety of bacteria.

Availability of extensive on-hand individual concentration data in normal weight individuals made recently possible a physiological based pharmacokinetic modeling and simulation (PBPK) study focused on children with obesity. According to the model used, these patients experience decreased weight-normalized clearance and volume of distribution of both drugs, so that these patients should require higher absolute doses under recommended pediatric weight-based dosing regimens. These data on one side fill the existing gap for TMP/SMZ [24] and on the other support Clindamycin’s current recommended TBW-based dosing [2, 21, 22, 24].

#### Other antibiotics

Currently, there are no conclusive data on other antibiotics that are widely used in clinical practice, such as clarithromycin or cefpodoxime [47]. Although some hypotheses can be generated on the basis of class analogies or the characteristics of metabolism and absorption, further studies in pediatric patients with obesity are needed to obtain a non-empirical guideline for these antibiotics [24].

#### Analgesics

Paracetamol (TBW dose 10–15 mg/kg/day every 4–6 h for age < 12y) is a widely used analgesic and antipyretic agent. Despite its widespread use, studies on pediatric population with obesity are scarce [26]. A pharmacokinetic study in children with obesity related non-alcoholic fatty liver disease (NAFLD) did not find significant differences in circulating acetaminophen blood concentrations after a single oral dose of 5-mg/kg (up to 325 mg) versus normal weight controls. Conversely, concentrations of the acetaminophen glucuronide metabolite in the plasma and urine of children with obesity related NAFLD were significantly higher [48]. Adults with obesity had significantly lower concentrations of acetaminophen after IV administration, thus exposing this population to a risk of therapeutic failure. Patients had higher concentrations of cysteine, mercapturate, and CYP2E1-mediated acetaminophen metabolites, demonstrating a risk of toxicity. Thus, individuals with obesity may not tolerate the higher doses required [2]. As also hepatic glucuronosyltransferase (UGT) activity is upregulated in the presence of hepatic fat, further studies on acetaminophen liver clearance are needed, especially when multiple doses are involved [5]. All in all, obesity-specific pediatric dosing guidelines for paracetamol are urgently needed [48]. N-acetylcysteine (NAC) is the antidote to paracetamol poisoning, but currently there are no data to support its use for patients > 100 kg. A recent study of patients with obesity treated with this antidote has shown that increasing the dose did not cause liver damage, defined as an AST or ALT ≥100 IU/L [49].

There are no data in the literature regarding ibuprofen, ketorolac, and ketoprofen in pediatric population with obesity. This represents a serious lack in light of the wide use of these drugs, especially ibuprofen.

Opiate agonists: Fentanyl (TBW dose 1–2 μg/kg/dose IM) and Morphine (TBW dose 0.10–0.15 mg/kg/dose every 4 h IM or 0.20–0.40 mg/kg/dose every 4 h OD) are opiate agonists primarily metabolized in the liver and eliminated intact or as metabolites in the urine; fentanyl is the most lipophilic. Although Ross et al. [28] recommended dosing Fentanyl with adjusted body weight (cofactor of 0.25), currently there is no agreement on the best body size descriptor for opioid agonists dosing in children with obesity. These drugs have a narrow therapeutic index and carry concerns regarding the risk of respiratory adverse events, particularly in the case of obesity. It seems, therefore, reasonable to exercise caution with empirically determined drug doses. As morphine is hydrophilic, itis recommended a TBW based dosing [28].

#### Anesthetics

Dexmedetomidine (TBW dose 1 mcg/kg) is a highly selective α2-agonist used for procedural and intensive care sedation which can also be used in pediatric intensive care as the second choice for the sedation of children during mechanical ventilation or sedation. Unlike propofol or fentanyl, it offers the advantage of not causing respiratory depression but retains potential cardiovascular side effects (i.e. hypotension and bradycardia), which require monitoring.

In adult patients with obesity, therapies with Dexmedetomidine result in higher plasma concentrations than in lean patients. Although Rolle et al. found that using lean body mass for scaling dosage is logical [50], the absence of large information on dexmedetomidine use in pediatric population has been a limitation. Only recently a study planned to identify the single dose needed to obtain sedation in 95% (ED95) of children with and without obesity has shown no differences in the dosage required for sedation in children suffering from obesity and those with normal weight [27].

Midazolam (TBW dose 0.1–0.3 mg/kg max 5 mg IV, IM) is a benzodiazepine used for anesthesia, procedural sedation, sleeping trouble, and severe agitation. Studies of midazolam pharmacokinetics in children with obesity have shown increased Vd in peripheral tissues and have advised an initial drug dose administration for continuous infusion higher than the standard dose to achieve therapeutic exposures [29]. Status epilepticus guidelines where the midazolam dose is adjusted to TBW or age, should take into account that a) this drug clearance increases as TBW increases, and b) clearance in teenagers is higher than in adults [5]. Gade et al. described the pharmacokinetics of midazolam with a two-compartment model. The rate of distribution was faster, and the peripheral volume of distribution was larger in adolescents with a high body mass index standard deviation score compared with adolescents with a lower standard deviation score. Simulations revealed that long-term infusions based on TBW could lead to high plasma concentrations in adolescents with obesity. Furthermore, simulated plasma concentrations after a fixed buccal dose indicated that adolescents with obesity may be at risk of sub-therapeutic midazolam plasma concentrations. The current dosing guidelines for status epilepticus, where the midazolam dose is adjusted to TBW or age, may lead to supra- and sub-therapeutic plasma concentrations, respectively, in adolescents with obesity [51].

Propofol (TBW dose 1–2 mg/kg pro dose) is a short-acting medication that results in a decreased level of consciousness and lack of memory for events. Considering the drug clearance, TBW has been suggested to be the most significant determinant of dose maintenance [30]. Olutoye et al. reported that children with obesity require a lower weight-based dose for anesthesia induction than normal weight children [15].

#### Steroids

A recent study showed that almost one-quarter of children with asthma receiving steroids are prescribed following a guideline-nonadherent order (22.2%), with up to a two-fold higher prevalence among those with severe obesity. Authors underlined the urgent need for standardization of drug dosing guidelines for children with obesity to avoid the risk of harm [38].

#### Inhaled medications

Inhaled medications commonly used in children include anesthetics, corticosteroids (comprising those for the treatment of asthma), and bronchodilators.

In the case of inhalation anesthetics, the absorption is determined due the oil/gas partition coefficient and the blood/gas partition coefficient (i.e. the extent of absorption and rate of absorption, respectively). The induction rate is inversely related to the blood/gas partition. Increased cardiac output of patients with obesity negatively regulates the induction rate and promotes drug removal. This is associated with a short induction time for volatile anesthetics, including enflurane, sevoflurane, and halothane, which, at least in part, is due to a reduced blood/gas partition [16].

No studies have specifically investigated inhaled corticosteroid dosing. However, there is some clinical evidence that standard doses of inhaled asthma-control drugs are insufficient for children with obesity (e.g., poor symptom management and pulmonary function testing scores). In one of the largest pediatric clinical trials involving over 1000 children with obesity compared to 2000 normal weight asthmatic controls, asthma responsiveness to albuterol standard single-dose in terms of spirometry and clinical symptoms, and the need for addiction of long-acting β2 agonists were inferior for children with obesity compared to lean ones. Therapeutic failure from standard inhaled drug doses is likely also caused by an increased inflammatory response caused by obesity, which adds to the baseline inflammation of asthma [2, 14]. Since the treatment of asthma and other allergic conditions may require a frequent use of steroids, prednisone should be particularly monitored during chronic treatment for its adipogenic effects [10].

#### Antineoplastic drugs

The development of obesity as a common consequence of childhood cancer treatment and /or elevated BMI already at diagnosis may hamper the correct computation of chemotherapy doses [52]. Current recommendations for the administration of chemotherapeutic agents are based on a Body surface area (BSA). Though, few pediatric studies have examined whether this approach results in optimal drug distribution and concentration. A study evaluating the PK of busulfan in bone marrow transplant recipients revealed that in children with BMI > 85th percentile use of the PK dose resulted in better attainment of the target Area Under the Curve compared to use of the adjusted ideal body weight [53]. In adults, a recent review emphasized the importance of body composition, i.e. of fat distribution, and not just obesity, when considering the effects of increased BMI on chemotherapy efficacy and cytotoxic clearance [54]. In pediatric oncology, it will be important to understand how body composition, such as subcutaneous and visceral fat distribution, affects the PK of chemotherapeutic agents. Of note in this regard, children often gain fat but lose muscle mass (sarcopenic obesity).

Large prospective studies are needed to better elucidate the impact of obesity on clinical outcomes in children with cancer. For example, although obesity and related comorbidities are associated with increased risk of anthracycline-induced cardiotoxicity, there are scarce data on this drug dosing both in adults and in children [55]. As cardiotoxicity risk appears depending on pharmacokinetic variability and doxorubicin clearance in the very young is significantly lower than in older children, it has been suggested patient-centered dose-adjustment should be guided by doxorubicin clearance based on body surface area and age rather than the current disparate initial-dose selection tools [56].

#### Drugs prescribed for the treatment of obesity comorbidities

Children with obesity can be affected by a number of comorbidities that are much lesser frequent in children with normal weight. In fact, diabetes, hypertension, hypercholesterolemia, and NAFLD are the main clinical pathologies associated with childhood obesity requiring medications generally scarcely used in normal weight children.

Metformin (500 mg day, max 2 g/day) is the first-line medication for the treatment of type 2 diabetes, particularly in patients with overweight. It is also used off-label for the treatment of polycystic ovary syndrome. Metformin should be initiated in children aged 10 to 16 years at a dose of 500 mg daily and titrated up in increments of 500 mg every 1 to 2 weeks (maximum dose 2 g) [57]. Children with obesity may require higher doses to achieve plasma concentrations comparable to children without obesity [2]. Treatment should always begin with or include lifestyle modifications [57]. A recent simulations study using a pediatric physiologically based pharmacokinetic (PBPK) model support the use of adult doses of metformin in older children and adolescents with obesity [41] and confirm a previous population pharmacokinetic modeling study utilizing a non-linear mixed effects modeling (NONMEM) [29, 58].

If the metformin at maximum dosage is inadequate, it is recommended to use insulin in addition to metformin rather than increasing further the dosage to avoid the onset of side effects (namely, gastrointestinal diseases and fatigue) [5]. Thiazolidinediones and sulfonylureas are not labelled for use in children and have a small amount of data in this age group. The first class gives cause for concerns due to the cardiovascular side effects (e.g. rosiglitazone) or anemia and hypertransaminasemia (pioglitazone) seen in adults. Both classes of drugs moreover are burdened by gain of weight, which is not desirable in a person with metabolic syndrome [10, 57].

Liraglutide is a hypoglycemic drug analogous to glucagon-like peptide 1 (GLP 1). In addition to controlling blood sugar, this molecule has shown to have an important effect on weight loss. In 2019, the European Medicine Agency (EMA) expressed a favorable opinion on the use of liraglutide in children over 10 years of age with an indication of type 2 diabetes mellitus not correctly compensated with metformin.

More recently it has been shown that in adolescents between 10 and 17 years of age with type 2 diabetes mellitus, chronic liraglutide is effective also for BMI reduction between 12 and 17 years of age with obesity and at the end of a 56-week treatment observed a reduction in BMI in patients treated with liraglutide compared to those who had taken a placebo [59, 60]. In the USA and several other countries, there are now indications for the use of this drug also for the treatment of obesity in patients aged> 12 years and weighing > 60 kg) at the same dose of adults (i.e. 3 mg, s.c.). A population pharmacokinetic analysis showed similar exposures in adolescents and adults having similar body weight ranges; body weight was identified as the most important covariate affecting exposure [61]. Despite these encouraging results, daily subcutaneous injection, and side effects such as nausea, vomiting, and diarrhea must be considered. Newer options with weekly dosing appear near the corner [60].

Proton pump inhibitors (PPIs) [e.g. pantoprazole (dose 10–20 mg/day) and esomeprazole (20 mg/day)] are drugs that inhibit stomach acid secretion, useful in the treatment of gastroesophageal reflux in obesity. At present, in clinical practice, empiric dose escalation of PPI is prescribed for children affected by obesity. As the long-term increase in exposure to pantoprazole may provoke osteopenia and vitamin deficiency, the TBW method however does not appear a correct approach for chronic PPI therapies. PPI dosing in obesity should therefore be the same as for children with normal weight [5, 42].

Clinicians need further studies to understand whether this applies to pantoprazole only or other PPIs.

Angiotensin-converting enzyme (ACE) inhibitors and angiotensin II receptor blockers (ARBs) are drugs used to treat blood hypertension. The updated guidelines of the American Academy of Pediatrics showed that nearly 800,000 American children and adolescents are affected by hypertension, especially in the context of renal diseases and metabolic syndrome [62]. Hanafy et al. [31] conducted a retrospective cohort study of hypertensive children affected by obesity treated with an ACE inhibitor, or a calcium channel blocker (CCB) for renal disease-associated hypertension. The mean medication doses were similar among the groups that used these drugs. This study suggests that children with overweight and normal weight may have a similar reduction in blood pressure when given equal doses of an ACE inhibitor or an ARB. Sparse information is available on ACE inhibitors. It seems that these medications should be administered similarly in children with obesity and normal weight, starting with an empiric low dose [32], followed by the medication adjustment (TBW dose for ramipril is 0.05–0.15 mg/kg/day max 40 mg/day). Hanafy et al. [31] also studied CCBs such as amlodipine (TBW dose 0.1 mg/kg/day), short-acting nifedipine, and long-acting nifedipine administered at similar mean doses in both groups of children with overweight and normal weight. In multivariate analysis, obesity had a significant effect on systolic blood pressure response.

Statins Childhood obesity is frequently accompanied by hypercholesterolemia, a risk factor for developing cardiovascular disease later in life. According to the FDA, the use of statins in pediatric hypercholesterolemia should be limited to pravastatin, simvastatin, atorvastatin, and lovastatin. In recent years, the use of some of these molecules has been approved by FDA in patients as early as 8 years of age [63].

The choice of treating hypercholesterolemia in children should be guided by identification of the lower therapeutic dose to obtain low-density cholesterol values below the 95th centile (≤ 130 mg / dL or 3.36 mmol/L) [6]. Studies on statins have shown that patients who are carriers of polymorphisms in the gene encoding the hepatic transporter SLCO1B1 are at higher risk of myopathy than wild type. Due to this pharmacogenetic concern, especially about atorvastatin and simvastatin, cases of overtreatment should be avoided [36].

#### Vaccines

Children with obesity may have a lower immune response, but the clinical findings are conflicting [64]. Furthermore, as certain dietary regimens can enhance memory T cell function, it is possible to harness the underlying mechanisms in the design of novel vaccination strategies [65]. During the Coronavirus 19pandemic, the scientific community has been additionally attentive to vaccination. Although obesity is a risk factor for poor prognosis, there are currently no data indicating whether overweight has a different immune response to vaccines in pediatric patients [66, 67].

Eliakim et al. [68] found that the tetanus antibody levels in children with obesity were lower than in normal weight children, but all had values higher than 0.1 UI/L. They supposed that the reduced specific antibody response might be due either to mechanical factors such as lower relative vaccination dose, and reduced absorption from the injection site due to increased adipose tissue, or to reduced immune response because of chronic low-grade inflammation expressed by higher levels of interleukin-6. Minana et al. [69] found only a weak direct correlation between BMI and antibodies to hepatitis B surface antigen. Others found that children with overweight/obesity respond to inactivated trivalent influenza vaccine (TIV) administration with similar or even slightly higher titers than normal weight controls [70]. All in all, it appears that children with obesity may receive normal doses of vaccine, provided that a BMI based needle length for deltoid IM injection is used to avoid they receive subcutaneous/fat pad injections [71].

#### Low-molecular-weight heparins

Low molecular weight heparins (LMWHs) are water soluble and tend to accumulate in the blood plasma and highly vascular tissues such as kidneys and liver, with poor distribution in adipose tissue. A comprehensive updated review of their efficacy and safety data shows that proactive adjustment of enoxaparin dosage at baseline may be necessary in pediatric patients with obesity compared to children with normal BMI [39].

Although it is still the most frequently used anticoagulant in children, and data from adults suggest that obesity may change its dosing requirements, current warfarin dosing guidelines for pediatric patients do not account for obesity. Only one study showed that pediatric patients with obesity have an increased time to reach therapeutic INR value when traditional warfarin dosing guidelines are used [72].

#### Vitamin D

Prevalence of hypovitaminosis D is more pronounced among both adults and children with obesity. The requirements of vitamin D necessary to reach sufficient 25(OH)D levels in these populations remain poorly defined in spite of a growing body of research. Serum 25(OH)D concentrations of children with obesity vs. children with normal BMI show a twofold lower rise in response to a single oral dose of vitamin D [73]. Moreover, a randomized clinical study in children and adolescents with overweight showed that three different doses (600, 1000 or 2000 IU/day) of vitamin D3 over 12 months increased serum 25(OH)D3 in a dose-response manner. The highest percentages of patients with values ≥20 ng/ mL were seen only among the 2000-IU group, implying therefore the superiority in effectiveness of this dose in comparison to the lower ones [43]. No matter what, the question is still open [74].

#### Drugs causing further weight gain: antipsychotics

The list of drugs causing weight gain as possible side effect is large and its full description is, therefore, behind the scope of this review [75]. Given the increase in psychopathological comorbidities in patients with obesity and the bidirectional relationship between psychiatric disorders we will limit our survey to antipsychotic drugs (APDs). Medications approved for pediatric treatment of these disorders are several and include haloperidol, thioridazine, risperidone, aripiprazole, quetiapine, olanzapine, and ziprasidone. It has been suggested that knowledge of their pharmacokinetics available in adults cannot be automatically transferred to children and adolescents. As an example, regarding risperidone it seems that, unlike adults, clinicians prescribing this drug need to consider sex, age, and weight, but not BMI when adjusting daily doses [33].

While offering many benefits for multiple psychiatric disorders, APDs as a class pose a major threat in their tendency to raise adiposity and metabolic dysregulation in an already metabolically vulnerable population. Weight gain observed in children is similar to or greater than that seen in adults, but relative weight gain tends to be more frequently higher in younger children, especially in correspondence of the initial APD exposure [34]. As recently reviewed by Goicke [35] although APDs are nowadays commonly used to treat an increasing number of psychiatric disorders, there are few studies on their correct dosing and therapeutic drug monitoring. Studies suggest that immediate effects on glucose regulation may play a role in APD-induced metabolic dysfunction, a prudent approach to dosing (start low, go slow) and careful monitoring of patients’ metabolism appears important. Discontinuation attempts after long-term use can also be beneficial. Although combined behavioral, dietary, and pharmacological interventions may mitigate APD-induced metabolic side effects, novel agents that can more completely prevent or reverse ADP-related weight gain and metabolic abnormalities are needed.

## Conclusions

Our overview shows a restricted amount of studies focusing on the obesity-related modifications in PK parameters of drugs used in pediatric practice. To guarantee an optimal pharmacological therapy, in terms of efficacy and safety, it is necessary to further investigate such parameters that mainly affect the drug dosing also in children with obesity. This will need both laboratory and clinical studies such as those performed for testing new drugs in any other affection that is not fully understood.

Choosing the appropriate dose of drugs to be administered to pediatric patients can be complex, even when considering a normally growing child. In fact, not all children have the same characteristics at the same age. Given the relentless rise incidence in childhood obesity, the problem faced by clinicians is considerably even more complicated. Physiology, drug parameters, patient factors, and methodology need to be accounted for while studying the influence of obesity on pharmacokinetics in children [76]. Despite signs of progress in knowledge on pharmacodynamics, pharmacokinetics, and other aspects of pharmacology, one is generally prone to determine drug dosing bearing in mind only the weight of the child. However, the evidence hitherto examined shows that the approach to the as close as possible ideal therapeutic dose by avoiding drug toxicity should consider many factors influencing the choice and dosage of a drug in childhood obesity. These include body surface area, metabolic capacity, and characteristics of the drug (lipophilicity and hydrophilicity). Enzyme activity, such as UGT, which is upregulated in the presence of hepatic steatosis is relevant as well [49]. Another relevant aspect such as gender-based pharmacology data must be considered and would merit a specific overview. In fact, hormonal aspects are profoundly different especially during adolescence, with impacts on the course of fat deposition in males and females Further research is required to improve confidence in pharmacokinetic predictions and dosing recommendations in the targeted patient population, and thus to ensure safe and effective drug therapies [7, 8]. A recent work focused on the relationship between body fat percentage (BF%) calculated by demographics, bio-impedance, and x-ray absorptiometry in children with normal and excessive body weights. Although methods of BF% estimation varied in accuracy and precision, differences resulted insignificant compared with typical *between-subject variability* of PK parameters seen in clinical studies. Interestingly, while BF% variability was similar across BMI groups and in both sexes, girls had a higher fat percentage in all BMI groups. Hence, for drugs in which BF% is an important determinant of alterations in drug disposition, it was suggested that PK analyses can utilize the mean BF% found for each combination of sex and obesity stage or, alternatively, sex and obesity stage can be tested concurrently as categorical covariate [77]. In addition to sex-related aspects influencing pharmacokinetics and pharmacodynamics, pharmacogenetics is an emerging area of research. It will hopefully address both genetic and sex aspects discrepancies in drug response and overcome the ‘one size fits all’ dosing to avoid incorrect exposures and the females ‘greater risk of developing adverse drug reactions (ADRs). The inclusion of other variables such as obesity and developing age will be a further task [78]. Due to the frequent use of off-label drugs in pediatrics, ADRs to a particular molecule require special attention [79]. Although metabolism and elimination of drugs could be affected by ‘excess fat’ with a potential increase in ADRs [40], published literature concerning the ADRs in individuals affected by obesity is extremely scarce and sometimes conflicting. Morales et al. [80] by investigating the incidence of ADRs in hospitalized patients found in fact that excess weight seems to decrease rather than increase the incidence of these unexpected events.

In summary, pending shared guidelines for the use of most pediatric drugs in children with obesity, the treatments should be approached critically each time, carefully considering the characteristics of the drug and the specific patient.

## Data Availability

“Data sharing is not applicable to this article as no datasets were generated or analyzed during the current study”.

## Abbreviations

25(OH)D: 25-hydroxyvitamin D
BSA: Body surface area
ACE: Angiotensin-converting enzyme
ADRs: Adverse drug reactions
ALT: Alanine aminotransferase
AST: Aspartate aminotransferase
BMI: Body mass index
CCB: Calcium channel blockers
CYP2E1: Cytochrome P450 2E1
% fT > MIC: (concentration of the antibiotic in the blood, times above the minimum inhibitory concentration)
HBV: Hepatitis B virus
IBW: Ideal body weight
IM: Intramuscular
INR: International normalized ratio
IV: Intravenous
LBM: Lean body mass
LBW: Lean body weight
LMWHs: Low-molecular-weight heparins
MIC: Minimum inhibitory concentration
NAC: N-acetylcysteine
NAFLD: Non-alcoholic fatty liver disease
NONMEM: Nonlinear mixed-effect modeling
PK: Pharmacokinetics
PPI: Proton pump inhibitor
PTA: Probability of target attainment
SC: Subcutaneous
SLCO1B1: Solute Carrier Organic Anion Transporter Family Member 1B1
TBW: Total Body Weight
TIV: Trivalent influenza vaccine
UGT: Hepatic glucuronosyltransferase
Vd: Volume of distribution
WHO: World Health Organization
